# Forensic characterization and genetic polymorphisms of 19 X-chromosomal STRs in 1344 Han Chinese individuals and comprehensive population relationship analyses among 20 Chinese groups

**DOI:** 10.1371/journal.pone.0204286

**Published:** 2018-09-20

**Authors:** Pengyu Chen, Guanglin He, Xing Zou, Mengge Wang, Fuquan Jia, Huiru Bai, Jida Li, Jian Yu, Yanyan Han

**Affiliations:** 1 Center of Forensic Expertise, Affiliated Hospital of Zunyi Medical University, Zunyi, Guizhou, China; 2 School of Forensic Medicine, Zunyi Medical University, Zunyi, Guizhou, China; 3 Institute of Forensic Medicine, West China School of Basic Science and Forensic Medicine, Sichuan University, Chengdu, Sichuan, China; 4 Department of Forensic Medicine, Inner Mongolia Medical University, Hohhot, Inner Mongolia, China; 5 School of Public Health, Zunyi Medical University, Zunyi, Guizhou, China; Xiamen University, UNITED STATES

## Abstract

X-chromosomal short tandem repeats (X-STRs) may assist resolution of complex forensic kinship cases and complement autosomal and Y-chromosomal STRs in routine forensic practice and population genetics. In the present study, we investigated the allele/haplotype diversity and forensic genetic characteristics of 19 X- STRs in 206 Guizhou Han and 1344 Meta-Han Chinese individuals using AGCU X19 PCR amplification system. Population relationships within five Han Chinese population groups (1344 individuals), between Guizhou Han and other 19 Chinese reference populations belonging to four language families (5074 individuals), as well as between Meta-Han Chinese and other 15 minorities (3730 individuals) were performed using Reynolds’s, Nei’s and Fst genetic distances, principal component analysis (PCA), multidimensional scaling (MDS), Structure and Neighbor-Joining tree. Mean paternity exclusion chance (MEC) in Duos > 0.99999999453588 and in trios > 0.99999999999781, as well as power of discrimination (PD) > 0.99999999999980 in Guizhou Han on the basis of allele frequencies. Consistent high MECs and PDs can be observed in Meta-Han Chinese population based on both allele diversities of 19 markers and haplotype diversities of seven linkage groups (LG). DXS10135 and LG1 are the most informative and polymorphic in Han Chinese group. The comprehensive population comparisons reveal that Han Chinese is a homogenous population and has the genetically closer relationship with Hmong-Mien-speaking groups than Tibetan-Burman-speaking and Turkic-speaking populations. In summary, AGCU X19 PCR amplification system is highly polymorphic and informative in Guizhou Han and Han Chinese populations. The comprehensive population data from 20 Chinese populations analyzed in this study may be used as a reference Chinese frequency database of X-STRs for forensic casework applications.

## Introduction

Short tandem repeats (STRs), also known as microsatellites and composed of repeating 2–6 base pair motifs, are highly variable variants with the number of approximately 700,000 in the human genome, play a pivotal role in population genetics, anthropology, genetic genealogy and forensics. Previous studies revealed that STRs are associated with the susceptibility and morbidity of more than 30 Mendelian hereditary disorders [1] and other complex traits heritability via regulating DNA methylation and gene expression [2–5]. STRs are highly prone to mutations through the gain or loss of single repeat units under DNA replication and evolutionary pressures (such as UV exposure, hypoxia, limited food sources and cold in Tibetans) [1, 6, 7]. This mechanism namely called simple stepwise mutation model (SMM) [1, 6]. Accumulating mutation evidence from pedigree or population whole genome sequence studies showed that the average mutation rate of the STR locus generally exceeds that of point mutation (single nucleotide polymorphisms with 10^−8^) by several orders of magnitude and is approximately 10^−3^ to 10^−4^ mutations per generation [6, 8, 9].

X-chromosomal STRs (X-STRs) with the unique pattern of inheritance (father transmits it to daughter and mother transmits one of them to her offspring) can complement autosomal and Y-chromosomal STRs in forensic identity (predominantly in identification cases of missing person and mass disaster victim) and complex kinship analyses, especially in the deficiency and incestuous cases [10]. Recently, AGCU X19 amplification system (AGCU ScienTech Inc., Wuxi, Jiangsu, China) was specifically designed to facilitate the X-STRs into the applications of forensic routine cases. This system is a five-dye, multiplex that allows co-amplification and fluorescent detection of 19 loci belonging to seven linkage groups (LG), in which DXS10148, DXS10135 and DXS8378 comprise the LG1 [11, 12], DXS10159, DXS10162 and DXS10164 comprise the LG2 [13], DXS7132, DXS10079, DXS10074 and DXS10075 comprise the LG3 [14], DXS6809 and DXS6789 comprise the LG4 [15], DXS7424 and DXS101 comprise the LG5 [16], DXS10103, HPRTB and DXS10101 comprise the LG6 [12], and DXS10134 and DXS7423 comprise the LG7 [14]. This new X-chromosomal STR amplification includes eleven X-STRs included in the Investigator^®^ Argus X-12 Kit [17] and eight additional new selected loci [18]. The impact of the new generation X-chromosomal STR amplification system is contingent upon its forensic reference database construction and discriminative ability in the personal identification and parentage testing. Tremendous progresses have been made in exploring the genetic variations and establishing the forensic reference database of 12 X-STRs included in the Investigator^®^ Argus X-12 Kit in China [14, 17], while forensic information focused on 19 X-STRs included in the AGCU X19 kit in Chinese ethnically/geographically diverse populations keep largely underrepresented [19–29].Han Chinese, who traces a common ancestry to the initial Neolithic Huaxia agricultural confederation residing in Yellow River and shares and exchanges culture and language with non-Han Chinese population when Huaxia culture continuous expansion toward southern China, exceeds 1.3 billion in the world and 1.282 billion in China (2010 census) [30, 31]. China is a state of considerable cultural, linguistic, genetic, phenotypic diversity in the 960 square kilometers of land. There are at least seven languages families which comprise Sino-Tibetan, Tai-Kadai, Hmong-Mien, Altaic, Austroasiatic, Indo-European and Austronesian. Guizhou, located in the southwestern of China, is demographically one of China's most diverse provinces including Han Chinese, Miao, Yao, Yi and other minority groups. Han Chinese nowadays account for more than 60% of the population in Guizhou and are mostly the descants of the ancient Han soldiers, who massively moved into Guizhou during the 8th and 9th centuries in the Tang Dynasty (https://en.wikipedia.org/wiki/Guizhou).

Previous population genetic studies have been concentrated on forensic characterization and genetic polymorphisms of AGCU X19 system in the Chinese Uyghur, Hui, Tibetan, Yi, Gelao, Miao, Li, Kazakh, Xibe and Han [19–29]. However, genetic variants of the X-STRs in Guizhou Han remains uninvestigated. To get a more complete picture of human X-chromosomal STRs of in China (especially for Han Chinese population), we genotyped 206 Guizhou Han individuals using AGCU X19 system and merged our newly-generated dataset with four publically available datasets of Han Chinese populations from different administrative divisions [22, 25, 26] (Dataset Ⅰ referred to as Meta-Han Chinese population consisting of 1344 individuals). We also merged our dataset with data from other 15 populations belonging to other language families (Dataset Ⅱ comprises 5074 Chinese individuals) [20, 21, 23–25, 27–29] to investigate the genetic relationships between Han or Meta-Han Chinese population and other Chinese minority groups.

## Materials and methods

### Ethics statements

This study was specially approved (Approval No. (2014)-1-044) by the Biomedical Research Ethics committee of Zunyi Medical University. All subjects were kept informed of the purpose and signed the informed consent before taking part in sample collection. Each subject was confirmed the offspring of indigenous Han nationality and without consanguineous marriage with minority groups at least three generations.

### Samples, DNA extraction and quantification

Peripheral blood samples were collected from 206 unrelated Han Chinese individuals (104 females and 102 males) residing in Guizhou province, southwest China. We used PureLink Genomic DNA Mini Kit (Thermo Fisher Scientific) to extract and isolate human genomic DNA, and used an Applied Biosystem 7500 Real-time PCR System (Thermo Fisher Scientific) and Quantifiler Human DNA Quantification Kit (Thermo Fisher Scientific) to measure the DNA concentration on the manufacturer’s protocol. Finally, we diluted the DNA to 2.0 ng/μL and stored at -20°C until amplification.

### DNA amplification and genotyping

We genotyped 206 Guizhou Han individuals on the ProFlex 96-Well PCR System (Thermo Fisher Scientific) using the AGCU X19 kit (DXS8378, DXS7423, DXS10148, DXS10159, DXS10134, DXS7424, DXS10164, DXS10162, DXS7132, DXS10079, DXS6789, DXS101, DXS10103, DXS10101, HPRTB, DXS6809, DXS10075, DXS10074 and DXS10135) on the basis of the recommendations. We employed a total of 10 μL as the final PCR reaction volume, including 4 μL of reaction mix, 0.2μL of A-Taq DNA polymerase, 0.8 μL of template DNA, 2 μL of primers and 3 μL of sdH2O (sterile deionized H_2_O). The PCR conditions for 10 cycles (95°C for 2 min, 94°C for 30 s, 60°C for 1 min and 65°C for 1 min) and 20 cycles (94°C for 30 s, 59°C for 1 min and 72°C for 1 min) and followed a final extension for 30 min at 60°C and finally holding at 4°C for preservation. Capillary electrophoresis separation of amplified products was conducted on the Applied Biosystems 3130 Genetic Analyzers (Thermo Fisher Scientific, MA, USA) with the POP7^®^ polymer and a 36cm capillary array. GeneMapper ID-X v.1.4 software (Thermo Fisher Scientific) was utilized to analyze the electrophoretogram and assign the genotypes of 19 X-STRs.

### Data analysis

We separately calculated allele frequencies in the males, females and pooled Guizhou Han Chinese population (206 subjects) and Meta-Han Chinese population (1344 subjects) using the modified PowerStatesV1.2 spreadsheet (Promega, Madison WI, USA). Arlequin software (version 3.5.2) [32] was used to estimate the genetic differentiation between males and females, and calculate the p values of Hardy-Weinberg equilibrium (HWE) and linkage disequilibrium (LD), as well as estimate the observed heterozygosity (Ho) and expected heterozygosity (He) in Guizhou females and Meta-Han females. Haplotype frequencies of seven linkage groups were calculated using the direct count method. The forensic parameters of polymorphism information content (PIC) and paternity exclusion chance (MEC) in the Trios and Duos (MEC_Krüger [33], MEC_Kishida [34], MEC_Desmarais [35] and MEC_Desmarais_Duos [35]) were estimated using StatsX (Statistics for X-STR) v2.0 [36] and the ChrX-STR.org 2.0 database (http://www.chrx-str.org/). Gene diversities (GD) of X-STRs and haplotype diversity (HD) of seven linkage groups were calculated using Nei’s formula [37] as employed in previous Y-chromosomal STR variation analyses [38, 39].

We used the Reynolds’s and Nei’s pairwise genetic distances, as well as Fst and corresponding p values to estimate the genetic differences and similarities using the PHYLIP version 3.5 packages [40] and Arlequin software (version 3.5.2) [32]. We first compared the genetic relationships of Guizhou Han and other four Han Chinese populations from different geographical regions [22, 25, 26] as well as other 15 Chinese previously published minorities [20, 21, 23–25, 27–29]. And then we investigated the genetic relation between the Meta-Han and other 15 reference populations [19–27, 29]. Population structure within Han Chinese female populations was dissected using Structure v.2.3.4.21 software [41] with K ranging from 2 to 5 under 10 repetitions. We employed ‘correlated allele frequencies’ and ‘Admixture’ models with 100,000 steps of burn-in and 100,000 repetitions for the MCMC. Structure Harvester was used to select the optimized K [42]. To reconstruct the population relationship along linguistically, ethnically and geographically diverse divisions, CLUMPP v.1.1.222 [43] and Distruct v.1.1.23 [44] were used to visualize the genetic structure. Multivariate Statistical Package (MVSP) version 3.22 software [45] was used to conduct principal component analysis (PCA) on the basis of allele frequencies and IBM SPSS Statistics version 21 (SPSS, Chicago, IL, USA) [46] and Molecular Evolutionary Genetics Analysis version 7.0 (Mega 7.0) [47] were used to respectively perform multidimensional scaling plots (MDS) and Neighbor-Joining (N-J) tree on the basis the pairwise genetic distance matrixes.

### Quality control

The experiment was conducted at the Institute of Forensic Medicine, West China School of Basic Medical Sciences & Forensic Medicine, Sichuan University. Control DNA 9947A and sdH2O included in the AGCU X19 kit were chosen as controls for allele assignment. This laboratory has been approved the accreditation of ISO/IEC 17025 and CNAS (China National Accreditation Service for Conformity Assessment). Besides, our experiment followed the recommendations of the Scientific Working Group on DNA Analysis (SWGDAM) [48] and the guidelines focused on the population data publication [49] and X-STRs analysis [50].

## Results

China, composed of 56 officially recognized ethnic groups and a population over 1,404 billion, harbors substantial genetic, linguistic, physical, cultural and diversity [30, 31] (1.2 billion Hans, 10.5 million Huis, 10 million Uyghurs, 9.4 million Miaos, 8.7 million Yis, 6.2 million Tibetans, 1.4 million Lis, 1.4 million Kazakhs, 0.55 million Gelaos, 0.19 million Xibes, and others (https://en.wikipedia.org/wiki/Han_Chinese). Here, to implement X-STR typing into routine forensic practice and establish Chinese reference database as well as investigate genetic diversity and forensic characteristics of Han Chinese population, we newly generated 19 X-STRs data from 206 Guizhou Han subjects (**S1 Table**) and combined previously published 4868 genotypes [19–27, 29], the dataset from 20 Chinese populations belonging to four language families: Sino-Tibetan includes Sinitic branch (Han [22, 25, 26] and Hui [25]) and Tibeto-Burman branch (Tibetan [20, 25, 27], Yi [19]); Tai-Kadai (Gelao, Li); Hmong-Mien (Miao [29]); Altaic comprises Turkic (Uyghur [20, 21, 25], Kazakh [23]), Tungusic (Xibe [24]). To characterize the genetic diversity of Han Chinese population, we then obtained 1344 genotypes of 19 X-chromosomal STRs in Han Chinese population from four different geographical administrations (Guizhou: 206, Sichuan [22]: 201, Hainan: 155, Guanzhong [26]: 474 and South China [25]: 308). We assessed the allelic and haplotype diversity, and forensic characteristics of Guizhou Han and Meta-Han Chinese population.

We then combined in total of 3730 previously reported genotypes from 15 different populations [20, 21, 23–25, 27–29] to investigate genetic relationships within and between Han Chinese populations and other ethnic groups along ethnic, linguistic and geographical divisions, which consist of two Sinitic-speaking populations (191 Wuzhong Huis (44 females and 147 males) and 200 Huis (68 females and 132 males) from Ningxia Hui autonomous region), two Tai-Kadai-speaking populations (513 Gelaos (265 females and 248 males) from Guizhou province and 167 Lis (108 females and 59 males) from Hainan province), four Turkic-speaking population (300 Ili Kazakhs (151 females and 149 males), 220 Xinjiang Uyghur males, 233 Ili Uyghurs (139 females and 94 males) and 211 Korla Uyghurs (66 females and 145 males) from Xinjiang Uyghur autonomous region), one Hmong-Mien-speaking population (268 Zunyi Miaos from Guizhou province), one Tungusic-speaking population (179 Ili Xibes (92 females and 87 males) from Xinjiang Uyghur autonomous region), and five Sino-Burman-speaking populations (331 Liangshan Yis (198 females and 133 males), 199 Dujiangyan Tibetans (103 females and 96 males) and 235 Muli Tibetans (118 females and 117 males) from Sichuan province; and 270 Tibet Tibetan males).

### Hardy-Weinberg disequilibrium and linkage disequilibrium

We first evaluated the Hardy-Weinberg disequilibrium (HWE) and Linkage Disequilibrium (LD) of 19 X-chromosomal STRs in Guizhou Han Chinese population and investigated the genetic differences among the 104 females and 102 males. **S2 Table** presents the observed heterozygosity (Ho), expected heterozygosity (He) and the p values on the basis of distribution of He and Ho. The Ho values span from 0.4808 (DXS7423) to 0.9135 (DXS10135) with the average of 0.7556 and He values vary from 0.4760 (DXS7423) to 0.9194 (DXS10135) with the average of 0.7663. X-STR locus of DXS10101 (0.0123) is not found to be in HWE before Bonferroni correction (p = 0.05). No significant deviations are observed after applying the multiplex correction. As listed in **S3 and S4 Tables**, all pairs of markers except for DXS101-DXS10101 (0.0000) and DXS10134-DXS10135 (0.0001) in Guizhou Han females and DXS10103-DXS10101 (0.0000) and DXS10103-DXS10101 (0.0000) in Guizhou Han males are found to deliver statistically significant results after Bonferroni correction (p = 0.00029).

To validate the HWE and LD measures in a bigger population size, we subsequently replicated the analyses in a Meta-Han Chinese population. One new dataset consists of a total of 1344 Han Chinese individuals (525 females and 819 males) from five geographically distinct populations: 206 aforementioned Guizhou Han individuals, 201 Sichuan Han individuals (93 females and 108 males) in the western China, 474 Guanzhong Han individuals (222 females and 252 males) in the northern China, 308 southeastern Han individuals (106 females and 202 males), and 155 Hainan Han Chinese males in the southern China. Ho and He span from 0.4629 (DXS7423) to 0.8914 (DXS10148) and 0.4975 (DXS7423) to 0.9193 (DXS10135), respectively. DXS10079 (p = 0.0343) and DXS10101 (p = 0.0083) are found to be deviated from the HWE before adjustment for multiple testing of Bonferroni correction (p = 0.05) in this Meta-Han Chinese group (**S5 Table**), however no deviations are found after correction. Additionally, significant associations in the pairwise LD analyses are found in four pairs after Bonferroni correction (p = 0.00029): DXS10103-DXS10101 (0.0000) and DXS10159-DXS10164 (0.0000) in Meta-Han females (**S6 Table**) and DXS10103-DXS10101 (0.0000) and DXS10164-DXS10075 (0.0001) in Meta-Han males (**S7 Table**).

### Allelic diversity and forensic parameters of Guizhou Han Chinese

The Fst and corresponding p values of 19 X-STRs between females and males in Guizhou Han are presented in **S8 Table**, no gender differentiation is identified (p = 0.05). We estimated the allele frequencies and corresponding forensic parameters in the females, males and pooled population, including gene diversity (GD), polymorphism informative content (PIC), mean paternity exclusion index in duos (MEC_Desmarais_Duos) and trios (MEC_Krüger, MEC_Kishida, MEC_Desmarais), as well as power of discrimination in males (PD_Male) and females (PD_Female). A total of 188 alleles with the corresponding allelic frequencies spanning from 0.0048 to 06683 in females, 179 alleles with frequencies from 0.0098 to 0.6275 in males, and 205 alleles with allelic frequencies from 0.0032 to 0.6548 in pooled Zunyi Han population are found. Five alleles (12.3 (0.0032) at HPRTB, 15.2 (0.0032) at DXS7424, 16.1 (0.0032) at DXS10074, 17.3 (0.0032) at DXS10162 and 19.3 (0.0032) at DXS10074) are not observed in females but in males. Twelve alleles (7 (0.0032) at DXS10164, 15.3 (0.0032) at DXS10074, 18.2 (0.0032) at DXS10075, 23.2 (0.0032) at DXS10159, 27.2 (0.0032) at DXS10148, 29.3 (0.0032) at DXS10101, 30.1 (0.0194) at DXS10148, 30.3 (0.0032) at DXS0.0032, 34.2 (0.0032) at DXS10101, 35.2 (0.0032) at DXS10134, 37.3 (0.0194) at DXS10134, 41.3 (0.0032) at DXS10134) are observed in females but not in males (**S9–S11 Tables**).

**Fig 1:**
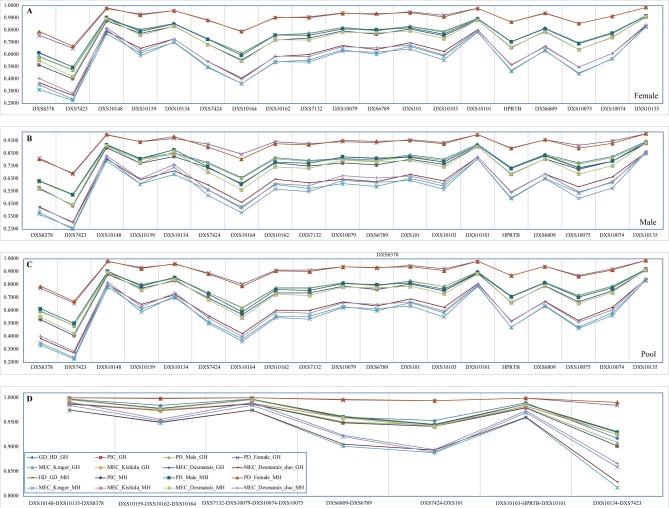
Forensic parameters of Guizhou Han and Meta-Han Chinese population. Parameters were calculated on the basis of allele frequencies in females (**A**), males (**B**) and pooled population (**C**). Forensic parameters estimated based on the haplotype frequencies in males (**D**).

**Fig 1 and S12 Table** present the forensic parameters. The maximums and minimums are consistently observed at loci of DXS10135 and DXS7423, respectively. In pooled Guizhou Han, HD and PIC span from 0.4848 to 0.9201 and 0.4075 to 0.9112, respectively. The PD values vary from 0.4832 to 0.9171 in males and from 0.6572 to 0.9872 in females. The MECs in duos range from 0.2726 to 0.8427 (MEC_Desmarais_Duos) and in Trios span from 0.2278 to 0.8326 (MEC_Krüger), 0.4073 to 0.9111 (MEC_Kishida) and 0.4075 to 0.9112 (MEC_Desmarais). Besides, high combined MEC_Desmarais_Duos, MEC_Kishida, MEC_Krüger, PD_Female, PD_Male are achieved as > 0.99999999453588, > 0.99999999999782, > 0.99999999999781, > 0.99999996992057, > 0.9999999999999999999996 and > 0.99999999999980 (**Table 1**).

**Table 1:** The combined forensic efficacy of AGCU X19 system in Han Chinese population.

Forensic Parameters		Guizhou Han	Meta-Han
PD_Male	Females	0.99999999999980	0.99999999999981
PD_Female		0.9999999999999999999957	0.99999999999999999999951
MEC_Krüger		0.99999996992057	0.99999997157916
MEC_Kishida		0.99999999999781	0.99999999999793
MEC_Desmarais		0.99999999999782	0.99999999999789
MEC_Desmarais_Duos		0.99999999453588	0.99999999472597
PD_Male	Males	0.99999999999981	0.99999999999979
PD_Female		0.99999999999999999999967	0.99999999999999999999951
MEC_Krüger		0.99999997185100	0.99999996804817
MEC_Kishida		0.99999999999800	0.99999999999760
MEC_Desmarais		0.99999999999802	0.99999999999761
MEC_Desmarais_Duos		0.99999999480885	0.99999999417952
PD_Male	Pooled groups	0.99999999999985	0.99999999999981
PD_Female		0.99999999999999999999974	0.9999999999999999999996
MEC_Krüger		0.99999997593596	0.99999997080635
MEC_Kishida		0.99999999999834	0.99999999999785
MEC_Desmarais		0.99999999999834	0.99999999999787
MEC_Desmarais_Duos		0.99999999560154	0.99999999468275
PD_Male	Haplotype	0.99999999998132	0.99999999999915
PD_Female		0.99999999999999999997	0.999999999999999999999933
MEC_Krüger		0.99999999742339	0.99999999960026
MEC_Kishida		0.99999999997377	0.99999999999113
MEC_Desmarais		0.99999999997663	0.99999999999898
MEC_Desmarais_Duos		0.99999999802622	0.99999999990756

PD, power of discrimination; MEC, mean paternity exclusion chance

### Allelic diversity and forensics parameters of Meta-Han Chinese

In our genetic diversity and forensic characteristic analyses of Meta-Han Chinese population, as shown in **S13 Table**, there is no gender differentiation in the Meta-Han Chinese population. Thus, allele frequencies and corresponding forensic parameters among females, males and pooled population are estimated subsequently. There are 261 alleles with the corresponding allele frequencies spanning from 0.0010 to 0.6381 in females, 258 alleles with allele frequencies varying from 0.0012 to 0.6264 in males and 293 alleles with the allele frequencies ranging from 0.0005 to 0.6330 in pooled Meta-Han population (**S14–S16 Tables**). A total of 8 alleles are not observed in 819 males: 23.2 (0.0011) at DXS10159, 42 (0.0005) at DXS10134, 18.2 (0.0005, 0.0005 and 0.0037) at DXS10162, DXS10103 and DXS10075 respectively, 32.3 (0.0011) at DXS10135, 14.2 (0.0011) at DXS10075 and 18.1 (0.005) at DXS10162, are only observed in the females. Similarity, 15 alleles in 525 females are not observed: 18.3 (0.0005) at DXS10074, 19.3 (0.0005 and 0.0005) at DXS10162 and DXS10074 respectively, 25.2 (0.0005) at DXS10159, 26.2 (0.0005) at DXS10101, 28.3 (0.0005) at DXS10148, 15.2 (0.0005) at DXS7424, 16.1 (0.0005) at DXS10074, 17.3 (0.0011) at DXS10162, 34.1 (0.0005) at DXS10134 and 34.1 (0.0005) at DXS6809, 35.1 (0.0005) at DXS10134, 36.2 (0.0005) at DXS10135, 41 (0.0005) at DXS10134 and 43.3 (0.0005) at DXS10134. A total of 261 alleles with corresponding allele frequencies varying from 0.0010 to 0.6381 in Meta-Han females, 258 alleles with corresponding allelic frequencies spanning from 0.0012 to 0.6264 in Meta-Han males and 293 alleles with corresponding allelic frequencies ranging from 0.0005 to 0.6330 in Meta-Han pooled population are observed. DXS8378 and DXS7423 are the less polymorphic loci with only 6 alleles are found in the 1344 Meta-Han Chinese individuals and DXS10135 is the most polymorphic and informative locus with 32 alleles observed.

**Fig 2:**
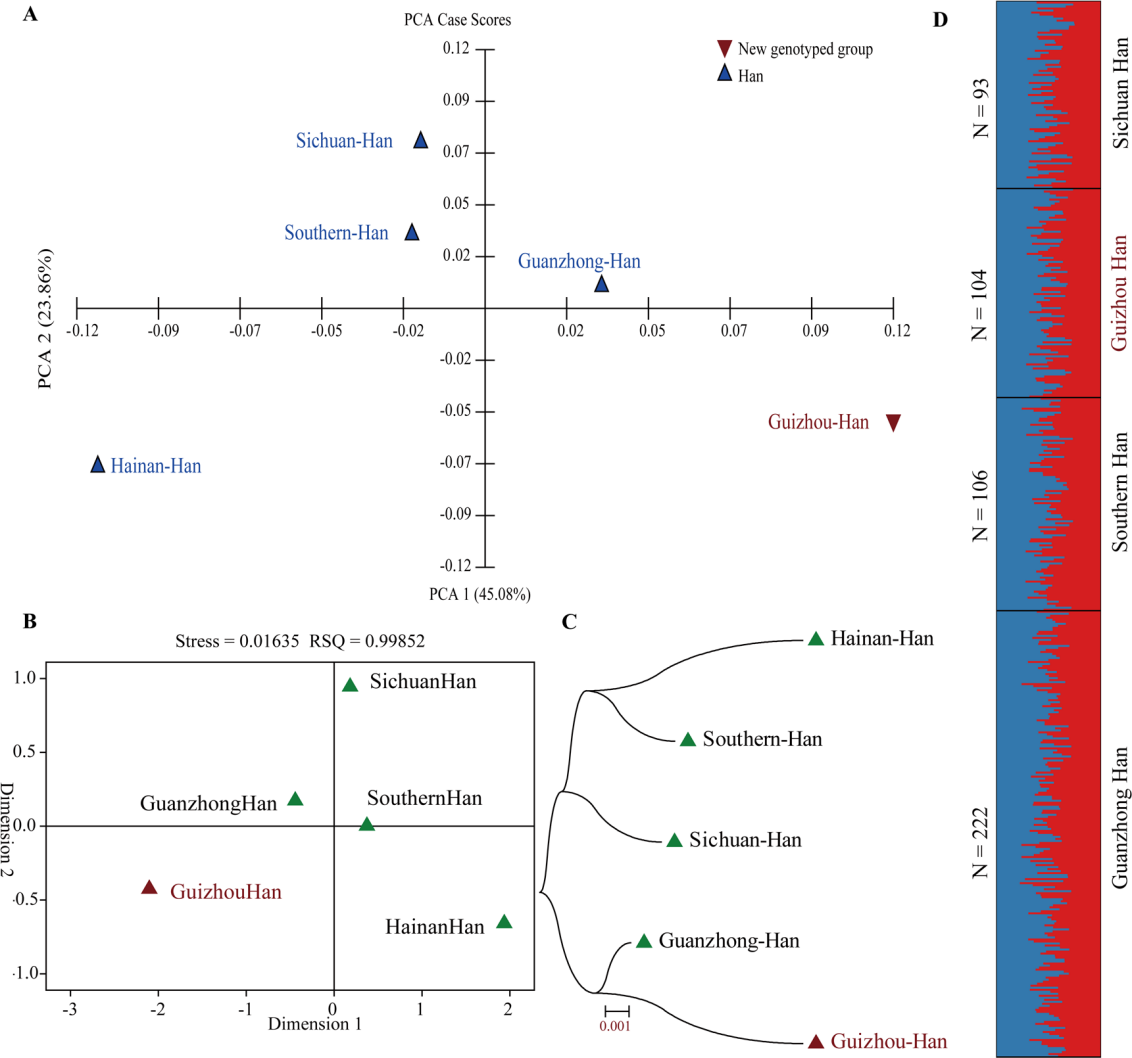
Genetic structure and population relationship between Guizhou Han and other four Han Chinese population. (**A**) Principal component analysis (PCA) revealed the genetic relationship on the basis of the first two components; (**B**) Multidimensional scaling plots showed the Han population relationship on the basis of Nei’s genetic distances; (**C**) A Neighbor-Joining tree reveled the phylogenetic relationship of Han Chinese populations; and (**D**) Genetic structure revealed by Structure among Han female populations.

As shown **Fig 2 and S17 Table**, GDs span from 0.4975 in Meta-Han females to 0.9202 in Meta-Han pooled population and PICs vary from 0.4186 in Meta-Han females to 0.9148 in Meta-Han males. PDs vary from 0.6686 in Meta-Han females to 0.9881 in Meta-Han males and 0.4971 in Meta-Han females to 0.9204 in Meta-Han males respectively focused on the forensic female population (PD_Female) and male population (PD_Male). The mean paternity exclusion chances in duos vary from 0.2826 in 525 Han females to 0.8484 in 819 Han males and in trios span 0.2358 in females (MEC_Krüger) to 0.9150 (MEC_Kishida). The combined MEC_Desmarais_Duos, MEC_Kishida, MEC_Krüger, PD_Female, PD_Male are achieved as lager than 0.99999999417952, 0.99999999999761, 0.99999999999760, 0.99999996804817, 0.9999999999999999999995, and 0.99999999999979, respectively (**Table 1**).

### Haplotype diversity

19 X-chromosomal STRs can be grouped into seven linkage groups on the basis of physical distances, previously linkage analyses and population genetic researches. The haplotype distributions of Guizhou Han population are presented in **S18 Table**, a total of 90, 61, 90, 34, 37, 68, 24 different haplotypes of LG1-7 are observed in 102 males, in which 79 in LG1, 39 in LG2, 79 in LG3, 11 in LG4, 17 in LG5, 47 in LG6, 9 in LG7 are unique. The most common haplotypes are 24.1-23-10 (3, 0.0294) in LG1, 24-19-10 (7, 0.0686) in LG2, 15-19-16-17 (3, 0.0294) in LG3, 33–16 (10, 0.0980) in LG4, 15–24 (14, 0.1373) in LG5, 16-13-32 (5, 0.0490) in LG6 and 36–15 (18, 0.1765) in LG7. The HD values are larger 0.95 with the exception of LG7 (0.9177). Forensic parameters on the basis of genetic variation of the single locus are listed in **S19 Table.** PICs vary from 0.9020 to 0.9876. The PD_Male and PD_Female are respectively span from 0.9087 to 0.9877 and 0.9850 to 0.9997. The MECs in duos span from 0.8290 to 0.9756 and MECs in trios vary from 0.8178 (MEC_Krüger) to 0.9876 (MEC_Desmarais). The combined values of PD_Male, PD_Female, MEC_Krüger, MEC_Kishida, MEC_Desmarais and MEC_Desmarais_Duos are 0.99999999998132, 0.99999999999999999997, 0.99999999742339, 0.99999999997377, 0.99999999997663 and 0.99999999802622, respectively.

In Meta-Han Chinese males (819 males), there are 394, 164, 394, 67, 69, 235 and 51 different haplotypes are found, in which 215 in LG1, 65 in LG2, 225 in LG3, 14 in LG4, 20 in LG5, 112 in LG6, 16 in LG7 are unique (**S20 Table**). The most common haplotypes are 26.1-24-10 (10, 0.0122) in LG1; 24-18-10 (49, 0.0598) and 25-18-10 (49, 0.0598) in LG2; 14-20-17-17 (12, 0.0147) and 15-19-18-17 (12, 0.0147) in LG3; 34–16 (71, 0.0867) in LG4; 16–24 (92, 0.1123) in LG5; 16-13-31 (50, 0.0611) in LG6; and 36–15 (105, 0.1282) in LG7. The HD and PIC values vary from 0.9310 to 0.9971 and 0.9258 to 0.9958, respectively (**S21 Table**). The values of PD_Male and PD_Female span from 0.9299 to 0.9958 and 0.9910 to 1.0000, respectively. The MEC values on the basis of formula devised by Krüger, Kishida, Desmarais for trios and Desmarais for duos vary from 0.8594 to 0.9851, 0.9254 to 0.9893, 0.9258 to 0.9958 and 0.8668 to 0.9917, respectively. The combined power of discrimination in males and females are 0.99999999999915 and 0.9999999999999999999999, respectively. The cumulative mean paternity exclusion chance in duos is 0.99999999990756, and the cumulative MECs in trios are larger than 0.99999999960026.

### Intra-population genetic differentiation among Han Chinese

To explore the genetic homogeneity and heterozygosity among Han Chinese populations along different administrative divisions, we calculated the Nei’s genetic distances between Guizhou Han and other four Han Chinese populations (**S22 Table**). Guizhou Han is genetically close to Guanzhong Han (Nei’s genetic distance: 0.0104), and keeps a relatively distinct genetic relationship with Hainan Han which is the southernmost Han Chinese population (0.0236). Population differentiation within five Han subpopulations is further dissected and visualized using principal component analysis, multidimensional scaling plot, Structure and one Neighbor-Joining tree (**Fig 2**). In the PCA dimensional plots constructed on the basis of PCA1 (45.08%) and PCA2 (23.86%), Guanzhong Han is located in the first quadrant near the X axis, Sichuan Han and South Han are located in the second quadrant near the Y axis. The remaining two groups are respectively located in the third quadrant (Hainan Han) and fourth quadrant (Guizhou Han) (**Fig 2A**). Consistent population distribution patterns are observed in the MDS based on the pairwise Nei’s genetic distances (**Fig 2B**). Phylogenetic relationship reconstruction reveals two genetically close clusters: one cluster comprises Guanzhong Han and Guizhou Han; and the other comprises Hainan, Southern China and Sichuan Han (**Fig 2C**). No population substructure is identified in the model-based genetic structure dissection (Structure in the **Fig 2D**). Genetic cluster analyses among Han Chinese populations show that Han Chinese are relatively homogeneity with the modest levels of genetic differentiation (average±standard deviation (sd): 0.0136±0.0044).

### Inter-population genetic differentiation among Guizhou Han and other 19 Chinese groups

**Fig 3:**
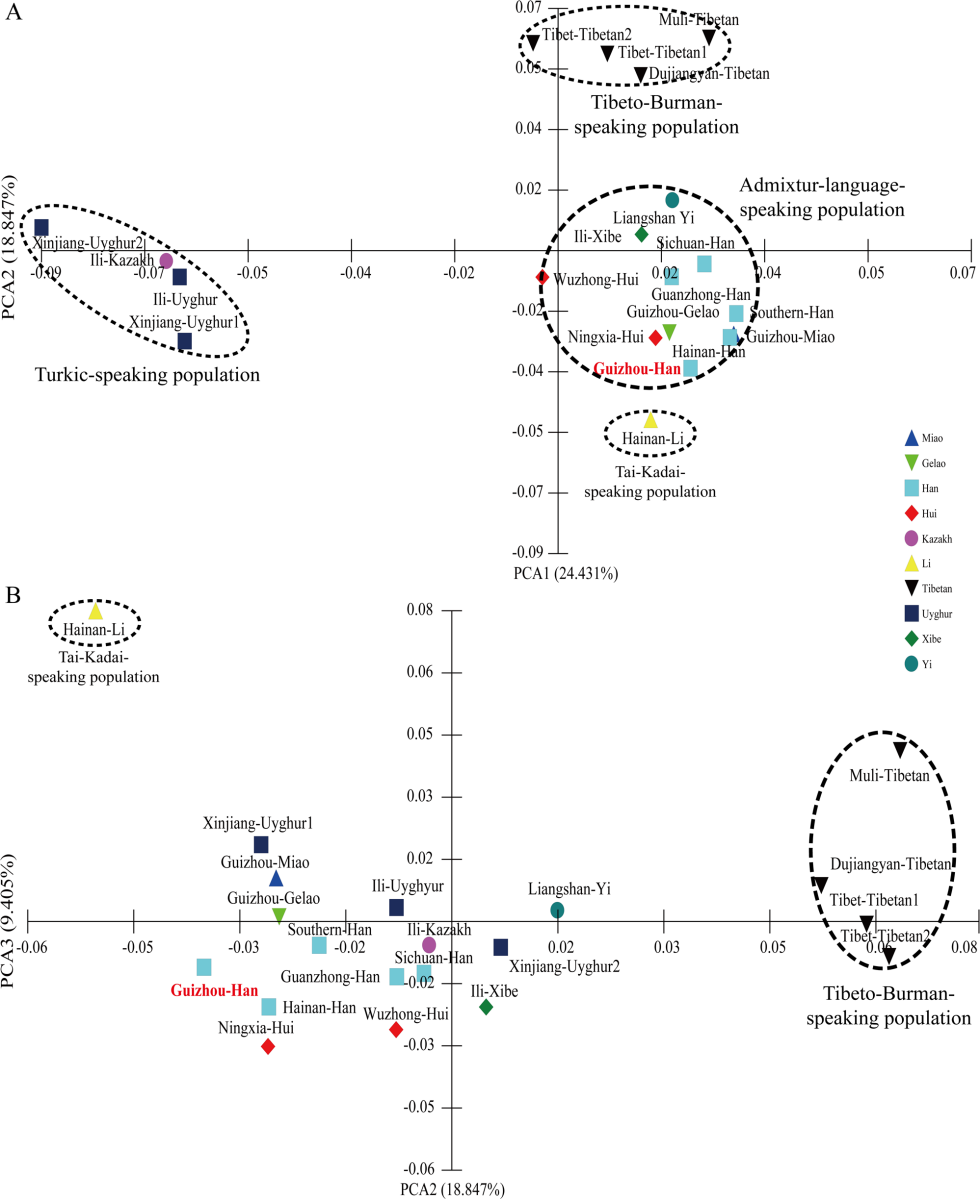
Principal component analyses between Guizhou Han and other 19 Chinese reference populations. (**A**) PCA was constructed on the basis of PCA1 and PCA2; and (**B**) Dimensional PCA plots were established according PCA2 and PCA3.

**Fig 4:**
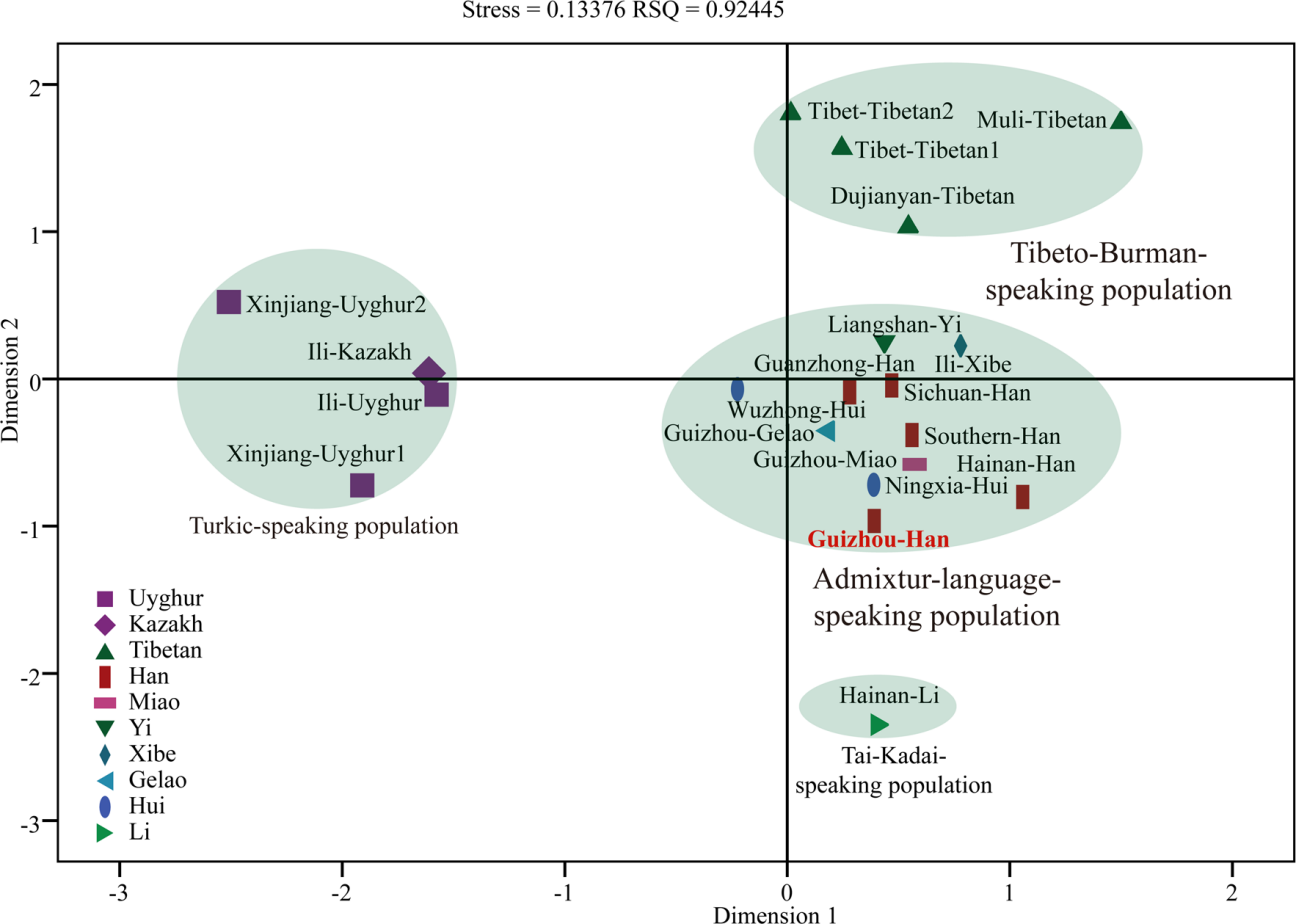
Multidimensional scaling plots showed the genetic relationship among 20 populations belonging to four language families.

**Fig 5:**
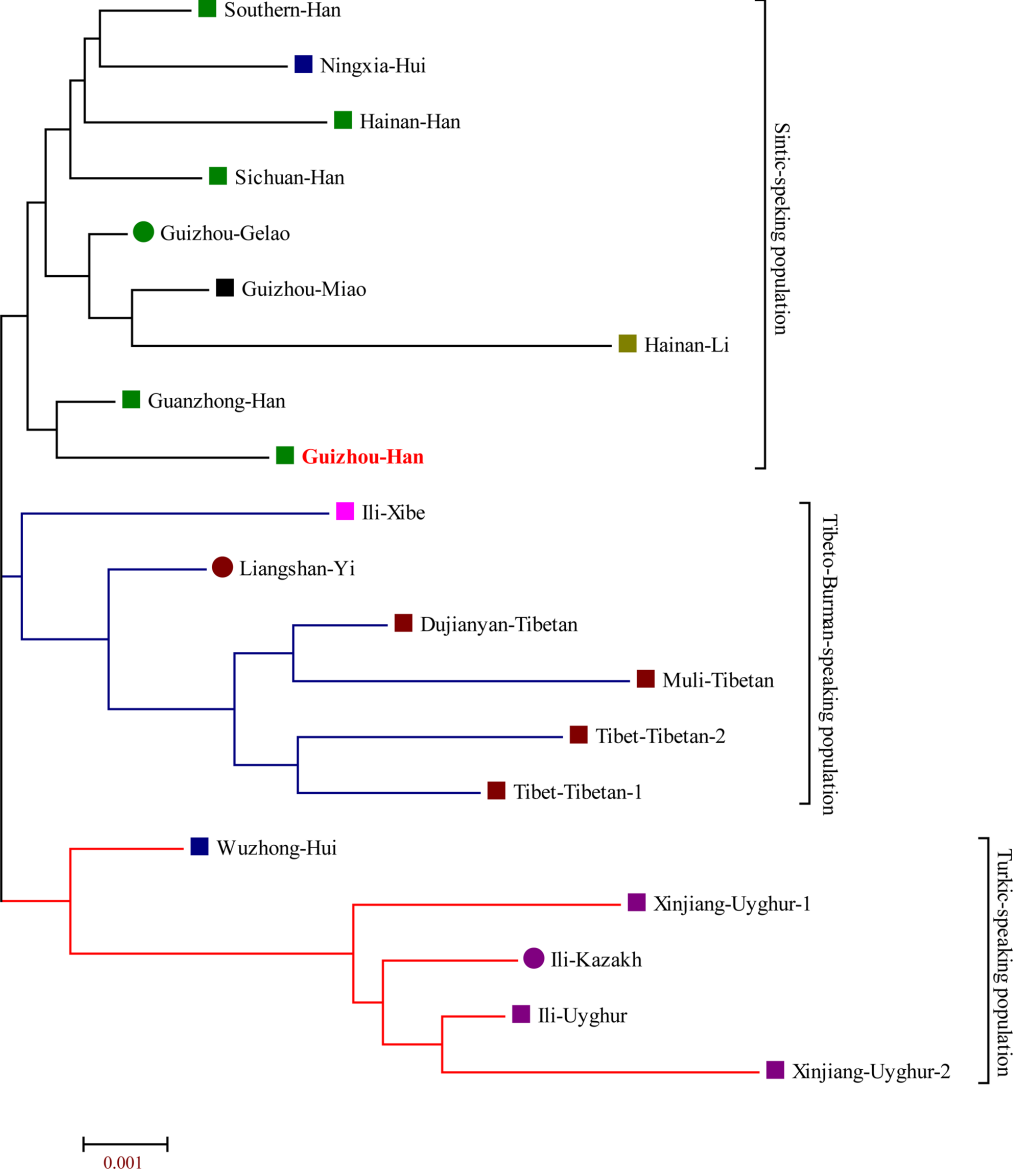
Phylogenetic tree showed the population relationship between Guizhou Han and other 19 reference populations on the basis of Neighbor-Joining algorithm.

The pairwise Reynolds’s genetic distances between Guizhou Han and other 19 Chinese adjacent populations are calculated on the basis of genetic variations of 19 X-chromosomal STRs and are listed in **S23 Table**. The Reynolds’s genetic distances range from 0.0022 (between Guanzhong Han and Guizhou Gelao) to 0.0161 (between Hainan Li and Xinjiang Uyghur2) whose average±sd is 0.0080±0.0032. Guizhou Han has the smallest genetic distance (0.0028) when compared with Guanzhong Han and has the largest genetic distance (0.0122) when compared with Xinjiang Uyghur2 with average±sd (0.0062±0.0027). The first ten PCAs can extract a total of 85.236% genetic variations from the 20 populations. **Fig 3** presents the population structure revealed by the combinations of PCA1 and PCA2, as well as PCA2 and PCA3 (PCA1: 24.431%, PCA2: 18.847% and PCA3: 9.405%). Four population clusters can be identified in **Fig 3A**: Turkic-speaking population cluster comprises three Xinjiang Uyghur populations (Xinjiang1, Xinjing2 and Ili) and one Ili Kazakh; Tibeto-Burman-speaking cluster consists of two Tibet Tibetan populations (Tibet1 and Tibet2), one Muli Tibetan and one Dujiangyan Tibetan; Tai-Kadai-speaking population cluster is made up of only one Hainan Li; and admixture-language-speaking population cluster comprises five Han Chinese population (Sichuan, Guanzhong, Guizhou, Hainan and South China) which speak Sinitic language, Liangshan Yi (Tibeto-Burman), two Sinitic-speaking Hui populations (Ningxia and Wuzhong), one Tai-Kadai-speaking Gelao (Guizhou) and one Hmong-Mien-speaking population (Guizhou Miao). PCA1 distinguishes Turkic-speaking population cluster from others, which locates on the left side of the X-axis in **Fig 3A**. And PCA2 differentiates Tibeto-Burman-speaking cluster, as well as Tai-Kadai-speaking population cluster from others, which respectively locate upside of the first quartile and the downside of fourth quartile, as well as other populations cluster together and locate intermediate of aforementioned two clusters. PCA3 can clearly separate Hainan li from other populations (**Fig 3B**). For further validation, we subsequently drew the MDS and N-J tree on the basis of Reynolds’s genetic distance matrix. Consistent population distribution patterns can be observed in the MDS analysis (**Fig 4**). In the N-J tree, Uyghurs, Kazakhs and one Wuzhong Hui form the nethermost cluster, Tibetans, Yis and one Ili Xibe form the intermediated cluster, and the remaining Hans, Lis, Miaos, Huis and Gelaos form the upper cluster. Guizhou Han first grouped with Guanzhong Han and then grouped with the other Sinitic-speaking population sub-cluster (**Fig 5**).

### Genetic relationship between Meta-Han Chinese and other ethnic groups

**Fig 6:**
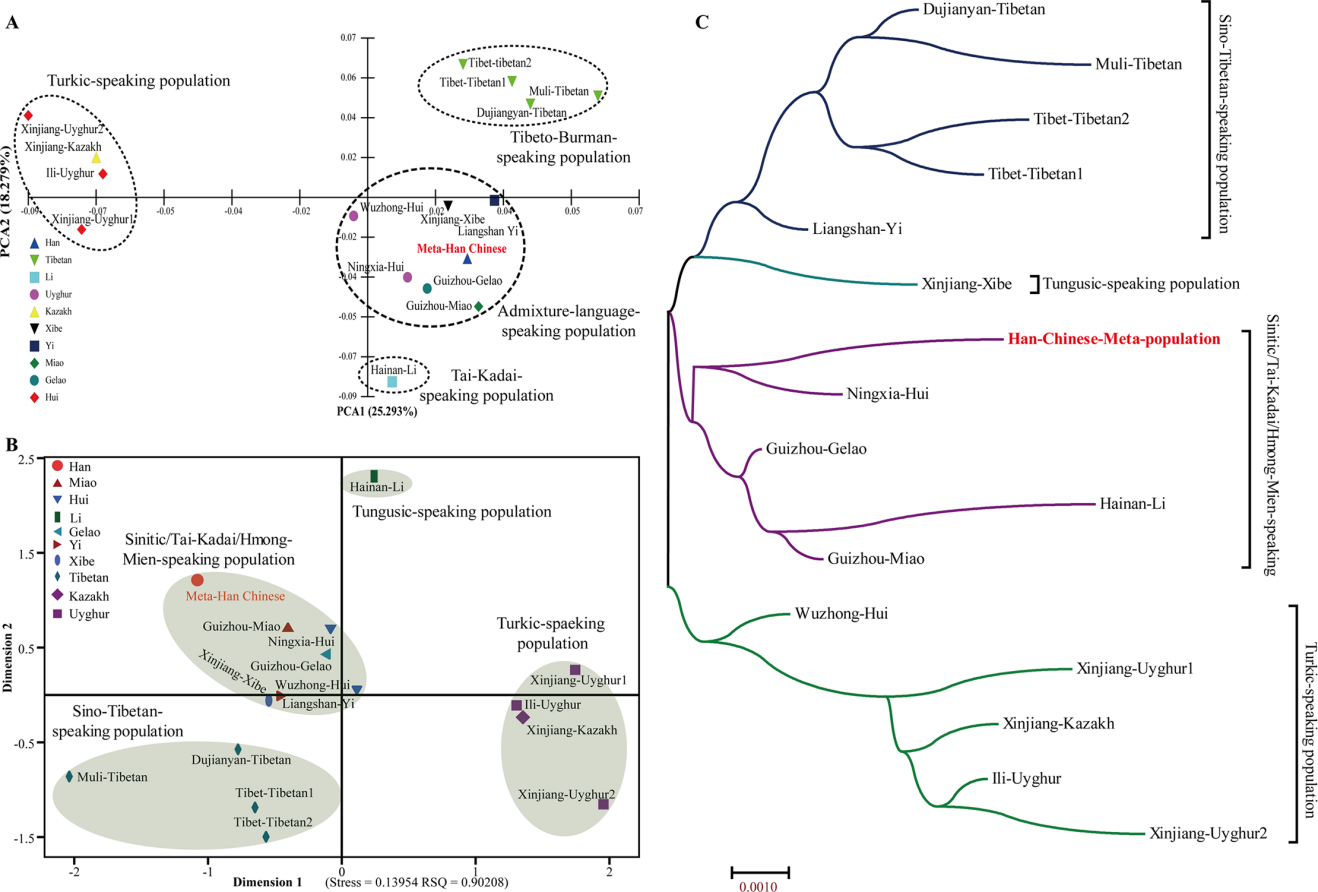
Genetic differences and similarities between Meta-Han Chinese population and other 15 reference populations. (**A**) PCA was constructed based on the first two components; (**B**) MDS was visualized on the basis of the pairwise Reynolds’s genetic distance matrix; (**C**) Neighbor-Joining tree showed the genetic homogeneity and heterogeneity between Han Chinese and Chinese minority groups.

Considering the homogeneity within Han Chinese populations, we integrated the 1344 genotype data from five different geographical divisions into one group as the Meta-Han Chinese population. Pairwise Reynolds genetic distances, PCA, MDS and N-J tree are performed to assess and dissect the genetic relationship between Meta-Han Chinese group and 15 Chinese relative populations. The first ten principal components (25.29%, 18.28%, 10.19%, 7.78%, 7.41%, 5.81%, 5.30%, 4.17%, 3.19% and 2.84%) from a national scale can extract a total of 90.265% genetic variation. **Fig 6A** was constructed on the basis of the first two components which reveals that the Meta-Han Chinese population constitute the same genetic group with admixture-language-speaking population cluster, suggesting there are a high level of gene flow between Han Chinese populations and other adjacent groups (Hui, Xibe, Yi, Gelao and Miao) and may have a common ancestry. As shown in **S24 Table**, the smallest genetic pairwise Reynolds’s genetic distance is observed between Meta-Han Chinese population and Guizhou Gelao (0.0067) and the counterpart is observed between Meta-Han and Xinjiang-Uyghur2 (0.0144) with the relative larger genetic heterogeneity (0.0104±0.0024) comparing with the overall genetic distances among all 120 pairs within 16 populations (0.0090±0.0030). In the MDS (**Fig 6B**) and phylogenetic tree reconstruction (**Fig 6C**) also consistently reveal that the Meta-Han group exhibits a closer affinity to other Sinitic/Tai-Kadai/Hmong-Mien-speaking populations. Collectively, we observe that genetic differences exist between Meta-Han group and Turkic/Sino-Tibetan-speaking populations and genetic similarities can be found between Han Chinese and Tai-Kadai/Hmong-Mien-speaking populations.

## Discussion

Clearly understanding the patterns of genetic variations of Han Chinese (the largest ethnicity in China and world) is important in the exploration of the population origin, migration, evolution and admixture in the prehistory and history, and providing investigative leadings and evidences in forensic cases. Although autosomal STRs have been the gold standard in forensic science and much effort has been made based on the genetic variations of autosomal or Y-chromosomal STRs in diverse populations [51–56], X-STRs have begun to draw more attention by forensic scientist with the appearance of Investigator^®^ Argus X-12 and AGCU X19 STR Kits. In this study, we first established one reference databases of Han Chinese population, extending already investigated data with additional 206 unrelated Chinese Han citizens and the total of 1,344 samples typed by AGCU X19 kit, to promote and implement X-STRs typing into Chinese routine forensic practice. Allele and haplotype frequencies and corresponding forensic parameters, as well as HWE and LD were first analyzed in the Guizhou Han and the comprehensive Han Meta-population. The combined MECs and PDs in Guizhou Han and Meta-Han Chinese combined with our previous studies [19, 22, 27] indicated the commutative forensic parameters of 19 X-STRs are high enough to meet the application of forensic complex biological relationship identification. 19 X-STRs PCR amplification system is discriminatory and informative for using as a complementary tool for autosomal, Y-chromosomal and mitochondrial genetic markers.

The peopling history of East Asia is complex [57, 58]. The comprehensive population comparisons (intra-population relationship among Han Chinese populations, between Guizhou Han and 19 national wide populations, as well as Meta-Han and 15 Chinese minorities) illustrate that they could better reflect linguistic, ethnical, geographical and historical relationships. Our results consistently demonstrate genetic affinity exists within linguistic/ethnical/geographical populations. Due to the complex origin, migration and admixture of Chinese populations, further studies based on high coverage whole genome sequencing of anatomically modern humans and Chinese ancient DNA are needed to promote the understanding of Chinese human evolutionary history and dissect the Chinese population structure as well as reconstruct the population genetic history.

## Conclusion

To implement 19 X-chromosomal STRs PCR amplification system into routine forensic practice, we genotyped 206 Guizhou Han Chinese individuals and combined with previously reported 4868 genotypes from 19 Chinese populations to extend and establish the reference database of Chinese populations along linguistic divisions. We used the Nei’s genetic distances, PCA, MDS and N-J tree to test the genetic homogeneity of the Han Chinese population from different geographical administrative divisions. Due to no significant genetic difference exists among them, we estimated the allele and haplotype frequencies as well as forensic parameters in Guizhou Han and Meta-Han Chinese population on the basis of allele frequencies and haplotype frequencies. DXS10135 and LG1 are the most informative and polymorphic in Han Chinese group. The cumulative power of discrimination and mean paternity exclusion chance according to the allele and haplotype diversity are high enough to complete autosomal and Y-chromosomal STRs in the forensic routine practices (complex kinship cases and individual identification) and population genetics. Subsequently, we compared Guizhou Han with 19 Chinese reference populations, as well as Meta-Han Chinese population and other 15 Chinese minority groups based on allele frequency distributions via pairwise Reynolds's genetic distances, PCA, MDS, and N-J tree. Population comparisons revealed the tight grouping within linguistic close populations of Tibetan-Burman, Turkic-speaking groups. Besides, Han Chinese is a homogeneous population and Guizhou Han and Meta-Han Chinese population have genetically close relationship with Tai-Kadai-speaking, Hmong-Mien-speaking populations. We concluded that the reference databases of AGCU X19 kit in Han Chinese populations, Tibetan-Burman-populations and Turkic-speaking populations are universally suitable and applicable for Chinese forensic casework.

## Supporting information

S1 Table Genotype data of 19 X-STRs in Guizhou Han population (n = 206, 104 females and 102 males). (XLSX)

S2 Table The heterozygosity and Hardy-Weinberg equilibrium testing of 19 X-STRs in Guizhou Han females (n = 104). (XLSX)

S3 Table The p values of the exact test of Linkage disequilibrium of 19 X-STRs in Guizhou Han females (n = 104). (XLSX)

S4 Table The p values of the exact test of Linkage disequilibrium of 19 X-STRs in Guizhou Han males (n = 102). (XLSX)

S5 Table The heterozygosity and Hardy-Weinberg equilibrium testing of 19 X-STRs in Meta-Han Chinese females (n = 525). (XLSX)

S6 Table The p values of the exact test of Linkage disequilibrium of 19 X- STRs in Meta-Han Chinese females (n = 525). (XLSX)

S7 Table The p values of the exact test of Linkage disequilibrium in Meta-Han Chinese males (n = 819). (XLSX)

S8 Table The Fst and corresponding p values between male and female in Guizhou Han population. (XLSX)

S9 Table Allelic frequencies of 19 X-STRs in Guizhou Han females (n = 104). (XLSX)

S10 Table Allelic frequencies of 19 X-STRs in Guizhou Han males (n = 102). (XLSX)

S11 Table Allelic frequencies of 19 X-STRs in Guizhou pooled Han population (n = 206). (XLSX)

S12 Table Forensic parameters of 19 X-STRs in Guizhou Han females, males and pooled population. (XLSX)

S13 Table The Fst and corresponding p values between males and females in Meta-Han Chinese population. (XLSX)

S14 Table Allelic frequencies of the 19 X-STR loci in Meta-Han Chinese females (n = 525). (XLSX)

S15 Table Allelic frequencies of the 19 X-STR loci in Meta-Han Chinese males (n = 819). (XLSX)

S16 Table Allelic frequencies of the 19 X-STR loci in pooled Meta-Han Chinese population (n = 1344). (XLSX)

S17 Table Forensic parameters of the 19 X-STR loci in Meta-Han Chinese females, males and pooled population. (XLSX)

S18 Table Haplotype and corresponding frequencies in Guizhou Han males (n = 102). (XLSX)

S19 Table Forensic parameters in Guizhou Han males on the basis of haplotype diversity (n = 102). (XLSX)

S20 Table Haplotypes and corresponding frequencies in Meta-Han Chinese males (n = 102). (XLSX)

S21 Table Forensic parameters in Meta-Han Chinese males on the basis of haplotype diversity (n = 819). (XLSX)

S22 Table Nei's genetic distances among 5 Han Chinese populations. (XLSX)

S23 Table Reynolds’s genetic distances between Guizhou Han and other 19 reference populations. (XLSX)

S24 Table Pairwise Reynolds's genetic distances between Meta-Han and other 15 populations. (XLSX)
